# Mothers’ Understanding of Infant Feeding Guidelines and Their Associated Practices: A Qualitative Analysis

**DOI:** 10.3390/ijerph16071141

**Published:** 2019-03-29

**Authors:** Andrea Begley, Kyla Ringrose, Roslyn Giglia, Jane Scott

**Affiliations:** 1 School of Public Health, Curtin University, Perth 6102, Australia; kyla.ringrose@curtin.edu.au (K.R.); jane.scott@curtin.edu.au (J.S.); 2 Telethon Kids Institute, Perth 6008, Australia; Roslyn.giglia@telethonkids.org.au

**Keywords:** infant feeding, introduction to solid food, complementary food, government guidelines, social construction

## Abstract

There is limited evidence to describe Australian mothers’ understanding of the Australian Infant Feeding Guidelines (AIFG). A qualitative inductive methodological approach was used in this study to explore experiences with the introduction of solid food. Seven focus groups with 42 mothers of children aged 4–18 months were conducted in disadvantaged areas in Perth, Australia. The mean age of infants was 9.6 months and mean age of introduction of solid food was 4.3 months (range 1.2 to 7.5 months). Almost half of the mothers in this study were aware of the AIFG however, only half again could correctly identify the recommended age for introducing solid food. Four themes and nine subthemes emerged from the analysis. Themes were (1) *Every child is different* (judging signs of readiness); (2) *Everyone gives you advice* (juggling conflicting advice); (3) *Go with your gut*—(being a “good” mother); and (4) *It’s not a sin to start them too early or too late* (—guidelines are advice and not requirements). The findings indicated that in spite of continued promotion of the AIFG over the past ten years achieving the around six months guideline is challenging. Professionals must address barriers and support enablers to achieving infant feeding recommendations in the design education materials and programs.

## 1. Introduction

The nutrition and health benefits of breastfeeding are well recognised and since 2003 the Australian Infant Feeding Guidelines (AIFG) have recommended mothers to exclusively breastfeed their infants to around 6 months, and this continues with the current edition [1,2]. Despite this being a relatively long-standing recommendation, studies have repeatedly shown that few Australian mothers achieve this AIFG recommendation and there are large variations in practice between women from different backgrounds [3,4,5]. The 2010 Australian National Infant Feeding Survey found that more than one half of infants (56.2%) had received solid food by five months of age and one quarter (28.4%) by four months of age [5]. The introduction of solid food before four months was more common amongst younger, less educated and low income mothers [5].

International and national infant feeding guidelines are an important means of communicating evidence-based best practice to health professionals, particularly child health nurses and these are then translated and communicated to new mothers through scheduled child health nurse visits for infants and targeted health promotion interventions. The evidence on the proportion of babies meeting the introduction of solid food recommendation suggest there are potential barriers and misconceptions to their uptake [6]. Research exploring the experiences in reality of applying the infant feeding guidelines will assist health professionals to understand these barriers and misconceptions.

Quantitative studies inform us that knowledge of the breastfeeding guidelines amongst pregnant Australian mothers is far from universal, with less than two thirds of expectant mothers being aware of them and, that even when aware of recommendations, four in 10 women intend, even in pregnancy, not to follow them [7]. There is limited evidence on how the interactions between influences on infant feeding knowledge and practices in order to understand the reasons mothers do not follow guidelines. Breastfeeding and the introduction of solid food occurs within societal expectations of norms and values constructed around government dietary advice [8]. Qualitative research is most useful to explore this complexity however, few studies of this kind have been conducted in Australia. Most qualitative research with mothers of infants has concentrated on breastfeeding decisions and indicate that the scientific and bureaucratic approach to breastfeeding by health professionals was not considered helpful compared to women’s expressed interests and their self-perceptions about being a good mother, particularly where advice was perceived as conflicting between health professionals [9,10,11]. 

A systematic review of qualitative studies by Harrison et al. published between 2000 and 2013 indicates three themes underpinning mothers’ decisions in transitioning from milk feeding to family foods as (1) an infant’s physical and behavioural cues, (2) maternal knowledge, skills and coping strategies, and (3) community pressure and inconsistent advice [12]. However this review included only one Australian study published in 2007 [13]. Another systematic review on infant feeding in the context of obesity by Matvienko-Sikar et al. included more recent Australian research of parents with children up to 2 years of age [14] and included four Australian studies carried out between 2010 and 2014, including one with Chinese immigrant mothers [15,16,17,18]. The review recommended that to better support parents with their decisions that infant feeding be considered as a changing process and consistent advice was required. Most recently, an Australian study sampled mothers from a mixed methods state-wide survey who had concerns about their infant’s weight and used individual interviews to explore reasoning in feeding decisions [19]. A number of key themes in response to weight concerns were found including the emotional response to expectations of being a responsible mother and being able to trust oneself and put trust in others in responding to cues from their babies. 

Trustworthiness is the term used to indicate quality in qualitative research and covers the systematic processes used (rigour) and the relevance of the results [20]. Studies conducted to date are of poor to moderate quality. The systematic reviews have shown inconsistent achievement of a number of reporting criteria indicating trustworthiness [12] using the consolidated criteria for reporting qualitative research (COREQ) checklist or they are rated moderate quality using the JBI QARI Critical Appraisal Tool [14]. Over half of the studies reviewed in the systematic review by Harrison et al. lacked description of the methodological approach and development of or use of theoretical perspectives to explain findings [21]. Clarity on the methodological approach will guide the sampling and subsequent procedural and analytical methods and in turn provide a justification for conclusions. 

Exploring how women’s knowledge of infant feeding practice is mediated by the social context or everyday life in which the practices exist and change as a result of AIFG and health promotion will assist in improving education [22]. Social constructionist theory has been used to assess how people interpret nutrition problems and recognises that there are conflicting and competing understandings of the phenomena of interest [23]. A key tenet in infant feeding is how risk related to the introduction of solid food is constructed from expert forms of knowledge given out by governments and health professionals which is often privileged over other forms of knowledge, such as a mother’s instincts and family advice [8]. The objectives of this research were to (1) identify women’s knowledge of infant feeding recommendations; (2) describe their actual infant feeding experiences; and (3) explore socio-ecological factors that determine women’s decisions and practices related to the introduction of solid food.

## 2. Materials and Methods 

### 2.1. Study Design

Mothers’ experiences and perceptions of introducing solid food was undertaken in Perth, Western Australia using a qualitative inductive approach [24]. Focus groups were chosen to address the study aim as this method is effective in promoting group interactions, sharing of experiences and debate that can clarify individual perspectives and produce insights of social norms [25]. In addition, qualitative research does not discriminate against people who have limited literacy and therefore are useful for collecting information from disadvantaged groups.

### 2.2. Participants

Recruitment used purposeful sampling to recruit mothers with recent feeding experiences who had introduced or were in the process of introducing solid food to ensure we captured a range of experiences (infants aged 4 to 18 months). Mothers were recruited primarily from existing community groups such as child health and community centres in socially disadvantaged suburbs. A flyer was distributed to these groups indicating University researchers would like to talk to mothers in a workshop about the trials and triumphs of infant feeding directly after their usual meeting the week before the focus group. These existing groups included both primiparous and multiparous women, as previous experiences with investigating attitudes to breastfeeding did not suggest that discussion was inhibited by the inclusion of women of mixed parity [26,27] and there was the advantage of the mothers being known to one another. Childcare was provided at all focus groups to limit distractions from infants and toddlers and all mothers present on the day participated. Participants were given a $25AUD gift voucher to recompense them in part for their time and out of pocket travel expenses.

### 2.3. Data Collection

A demographic questionnaire was developed incorporating questions on knowledge of the AIFG and actual age of introduction of solid food for their child. The focus group script was developed based on a review of the literature and feedback from a stakeholder reference group. The stakeholder reference group included state government and non-government experts from policy, nursing and dietetic backgrounds. The focus group script was piloted with a convenience sample of women closely resembling the intended target group recruited from a local childcare centre. Feedback from the pilot was used to modify and refine the script and provide an estimate for the duration of the session. This script ensured that discussions developed in line with the research objectives and allowed for comparison of information between focus groups. However, there was flexibility to enable exploration of emerging themes as the data analysis proceeded. The following focus group script topics were used for discussion:

- Solid food considerations before baby was born, age of starting
- Importance of introducing solid food
- Experiences of the first day, planning and emotions
- Information, events and infant behaviour used to direct personal practices
- Mother’s perception of her infant’s needs
- Interpretation of infant temperament and behaviour—signs of readiness for solid food
- People, events, barriers or facilitators which prevented or assisted the achievement of their feeding intentions (Mind mapping of influencers)
- Experiences with baby foods—suitability of foods and textures (texture photos prompt)
- Women’s awareness of current infant feeding recommendations and of the scientific rationale for the recommendations
- Personal beliefs about the appropriateness and relevance of guidelines

Discussion was encouraged and aided through the use of mapping [28] when brainstorming infant feeding information sources and influences. Sources of information were concept mapped onto a white board and then levels of influence determined using larger or smaller circles by participants. Photos of weaning foods were used (both homemade and commercial) to prompt memory and reduce misunderstandings about descriptions of food texture [29].

Focus groups were moderated by the two of the authors who have experience in conducting focus groups in this target group. An assistant moderator observed the discussion and took notes on both verbal and nonverbal communication. Focus groups lasted between 60 to 70 minutes and had between 4 to 8 participants and continued until saturation of concepts was achieved [30]. A number of steps central to high quality qualitative research [31] were employed to maximise the rigour and trustworthiness of the data collected throughout the research process. Firstly, a brief form of member checking occurred at the completion of each focus group when the assistant moderator offered a one to two minute summary of the discussion directing attention to those topics that are of critical concern to the study. Following the summary, participants were asked if the summary was complete and were given the opportunity to amend the summary. Immediately, following the departure of the participants, the moderator and assistant moderator spent 20–30 minutes reflectively developing field notes on what they considered to be the rich points of the interview and the most notable quotes, and compared and contrasted the interview with previous focus groups. The two moderators conferred over each new set of field notes as the groups progressed.

### 2.4. Ethical Considerations

All potential participants received a written and verbal description of the project including its aims and expected outcomes and were informed of their right to withdrawn at any time without prejudice. Curtin University Human Research Ethics Committee approved the study (SPH-40-13).

### 2.5. Data Analysis

Discussions were audio-recorded and then transcribed verbatim by a professional transcription service and checked twice for accuracy by the research team (dietitians). Analysis of focus groups commenced immediately after each group. Transcripts were managed using NVivo (Qualitative Solutions and Research Pty. Ltd., Melbourne, Australia) to store, code, retrieve and compare data from the focus groups. A social constructionist perspective provided the data analysis framework for identifying themes and assessing coherence of findings with past research findings.

The primary author used an inductive approach whereby the data were transcribed and checked for accuracy, initial readings of transcripts where conducted and notes about interesting points made. Then codes were assigned to sections of the data using descriptions from the data and to look for patterns (big ideas) emerging following processes suggested by Miles and Huberman (1994) [32]. Thematic analysis using a social constructionist approach was used to develop themes from patterns and connections between codes. Continued immersion in the data refined the themes and subthemes, and tested emerging codes as new data were integrated into the analysis. The research team used an ongoing reflective process of code and theme analysis between to confirm the results with a second author. Results were communicated with the stakeholder reference group. Quotations have been used to illustrate key themes and subthemes. All participants were assigned an alias representing focus group number and participant number based on order of speaking in the transcripts. The consolidated criteria for reporting qualitative research (COREQ) checklist has been applied to the description of methods and results [21].

## 3. Results

### 3.1. Participants

A total of seven focus groups were conducted involving 42 mothers. The characteristics of participants are reported in Table 1. The mean age of mothers was 30.3 years (range 22 to 44) and is similar to the average age of women giving birth in WA in 2013 of 29.8 years [33]. Women aged 35 years or older represented 26.2% of participants in this study compared to 20.5% in the same age group of women giving birth at the state level [33]. There were 42.9% of women with bachelor degree or higher level of education which compares with 35.7% in women of child bearing aged 20 to 40 year [34]. The mean age of the infants of the focus group participants was 9.6 months and mean age of introduction of solid food was 4.3 months (range 1.2 to 7.5 months). 

### 3.2. Knowledge of the Australian Infant Feeding Guidelines

Just under half of participants (46.3%) indicated they had heard of the AIFG [2] and less than half (43.9%) correctly identified around 6 months as the recommended age for introducing solid food. Of those who claimed to have heard of the AIFG only 10 of those 19 women (55.6%) correctly identified 6 months as the age at which solid food should be introduced. 

### 3.3. Themes and Sub Themes

**Figure 1:**
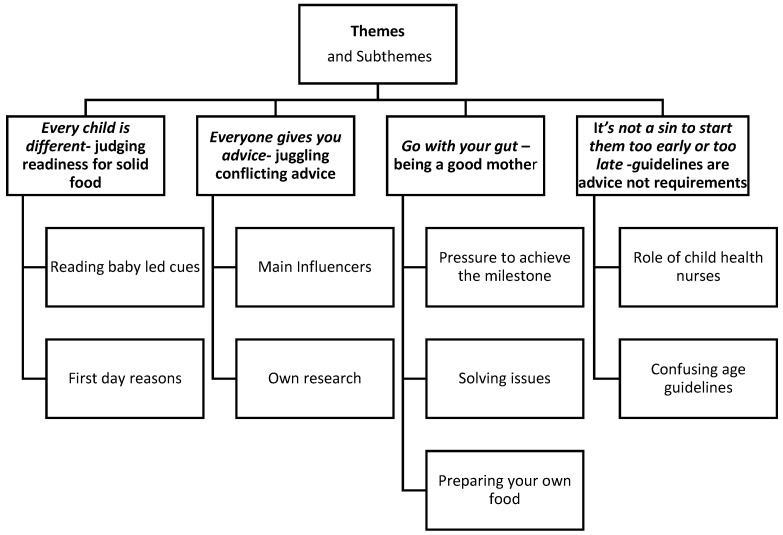
Introduction of Solid food themes and subthemes.

Four themes with nine sub-themes emerged from the analysis and are summarised in Figure 1. Themes identified included (1) *Every child is different*—(judging signs of readiness); (2) *Everyone gives you advice*—(juggling conflicting advice); (3) *Go with your gut* (being a ‘good’ mother); and (4) *It’s not a sin to start them too early or too late* (guidelines are advice and not requirements). 

#### 3.3.1. Theme 1: *Every child is different*—judging readiness for solid food

The discussion across all the groups communicated a strong image that babies controlled feeding decisions, indicated when they were ready and what they liked, and mothers interpreted these cues to justify a number of decisions around timing of solid food and types of foods to be introduced.

##### Subtheme—Reading Baby Led Cues

Mothers described a constant juggling of interpretation of, and reaction to, their baby’s cues and justifying feeding decisions:

They are this person, you have to kind of go with how the child reacts. Like it doesn’t concern me when (baby name) doesn’t eat something because I’m not going to eat everything and do you know what I mean she’s just a tiny person and she’s got her own what she likes and what she doesn’t like.

*(FG6 Participant 3)*

Readiness for solid food was mostly justified by babies showing increased interest in surroundings, particularly with watching closely parents or other children eat being considered a specific sign of readiness for solid food, irrespective of age:

‘cause she was doing the stare thing me and my husband I kept coming to him, should we do it, and he’d just go whatever you think and I’d go but I don’t know what do you think and he’d just go whatever you think. You know it says 4–6 months and we did it (introduce at 4 ½ months) because she’d stare at us when we eat in front of her.

*(FG2 Participant 6)*

Mothers when prompted described physical developmental signs such as the babysitting up unsupported as being a sign that their baby was capable of feeding themselves, but these did not form part of their decision-making. Baby-led weaning had entered the lexicon of mothers but this reference was primarily about reading signs from the baby on what they wanted, part of judging the readiness for solid food and finger food textures:

… saying that you know the food had to be led by the baby, so introducing food was always had to be after a feed and had to be you know led by the baby. So according to quantity, quantity, what they ate, their likes and dislikes, and something else I can’t even remember what it was but yeah.

*(FG5 Participant 3)*

##### Subtheme—First Day Reasons 

The day that solid food were introduced was described as exciting and stressful at the same time. Given that in many instances the actual first day of introducing solid food was opportunistic or ad hoc there wasn’t a major focus on the infant feeding guidelines during this process.

You know it was just, it was a spur of the moment kind of.

*(FG5-Patricipant 2)*

#### 3.3.2. Theme 2: *Everyone gives you advice*—juggling conflicting advice

Women had not considered the introduction of solid food prior to or during their pregnancy. Knowledge about this was gained in the first few months postpartum from multiple sources and the mapping activity and associated discussion highlighted the variability. 

##### Subtheme—Main Influencers

Significant others who consistently influenced the decision to introduce solid food included maternal grandmothers and other family members who had infant feeding experiences. Mothers most likely listened to their own mothers or other mothers as it was seen as advice in their best interests rather than trying to achieve health professional/guideline recommendations.

… that had a huge impact ‘cause then I felt like I had the approval of, their approval oh I’m not stupid in thinking that I should be doing this. So it was, yeah it was nice too.

*(FG5 Participant 3)*

Friends and other mothers were consistently mentioned as an influential source of experienced information, so worth listening too even if they said different things.

Whereas with my youngest one it was I don’t know if it was almost like if everyone else is doing it them I felt that I should be.

*(FG3 Participant 1)*

Partners were mentioned often with a humorous or dismissive tone as they often sourced their own advice and tried to override mothers with information they had gained from peers at work or read themselves.

Mine always wanted to feed him. I’d say no you’re not feeding him yet, ‘can I feed him now’, no not yet.

*(FG5-Participant 1)*

##### Subtheme—Own Research

Women talked about doing their own research (FG7 Participant 1) to address the perception that the advice was conflicting.

I started thinking about solids probably when she was a few months old, started doing that research and looking into the guidelines and trying to work out what sort of approach I wanted to take to it…

*(FG4 participant 1)*

Women had to decipher the advice and make up their own minds as they saw themselves as the key decision maker.

I’m just sick of all of the conflicting, like having to explain what I’m doing to everyone and why, like everyone just tries to tell me what to do. Like my husband will say why haven’t you given her this or why did you give her this or what are you doing with this and then I’ll try to explain to him and then my parents will say what are you doing, why are you doing this.

*(FG2 Participant 2)*

#### 3.3.3. Theme 3: *Go with Your Gut*—Being a Good Mother

This theme recognized the identity making in motherhood. Being a good mother required making decisions based on variable advice and caused some anxiety and concern. Justifying the importance of their role and decision-making dominated the focus group discussion.

You’re not doing it to harm your child I think that you’ve got that innate fear that you’re doing everything wrong as a mum you know….

*(FG4-Participant 2)*

My obstetrician told me to like go with my gut, like be, ignore a lot of, don’t over research, don’t over analyse, do what you’re most comfortable with.

*(FG7- Participant 2)*

##### Subtheme—Pressure to Achieve the Milestone

Moving onto solid food was an important developmental milestone, but came with trepidation.

Oh I think we were all very excited it was a big milestone. For me it was a big milestone, it’s like wow suddenly he can eat things what’s going on he can’t just have milk anymore he needs something else.

*(FG2-Participant 1)*

The majority of women interviewed had introduced solid food before six months with some even before four months. Women justified the timing for starting on solid food in a number of ways but there was a strong and consistent statement across the focus groups of peer pressure influencing the timing of introduction of solid food.

You want your child to be winning the race.

*(FG6-Participant 7)*

I just felt that a couple of girls in our mother’s group they just really wanted to push their babies anyway so they just couldn’t wait for them to crawl, couldn’t wait for them roll, it was just kind of generally speed up the process rather than just like let it happen so…

*(FG7-Participant 3)*

Online baby groups and social media provided subtle forms of peer pressure, with starting solid food being seen as a developmental milestone described as ‘*bragging stuff*’ (FG7 Participant 4). Whilst mothers engaged with these forums they realised their limitations as sources of advice and information.

You’re not hearing about the average child you’re hearing about the geniuses and the ones that, the extremes.

*(FG7-Participant 2)*

##### Subtheme—Solving Issues

Solid food was positioned as a panacea for a range of issues for the baby’s health and wellbeing. Mothers who introduced solid food early generally were seeking a solution to a real or perceived problem such as colic or infant sleeplessness. Insufficient weight gain as compared to norms for development resulted in some mothers being encouraged to introduce solid food early. This need for early introduction was communicated by a range of health professionals (child health nurses, paediatricians and general practitioners).

Mine started as a weight issue, we dropped off the scale and needed, were told basically to start…

*(FG5-Partcipant 3)*

The decision to introduce solid food early may be a coping strategy for families who think that this will alleviate issues with sleeping and provide a practical solution for family functioning.

You’re told that they will sleep through the night if you give them more solids during the day.

*(FG2 Participant 1)*

There were tensions in decisions over what foods to introduce to infants. Mothers wanted to provide good food that they could trust however this was interpreted from “cooking everything from scratch” through to preferring commercial foods due to a lack of confidence in own cooking.

I’m a terrible cook. I’m just, I’m not particularly good cook and although I did you know my own apple, mashed up apple and a few things like that just to give a bit more variety and to get the meat, because I didn’t know how to puree it…

*(FG7-Participant 2)*

##### Subtheme—Preparing Own Food

In some instances, women expressed a sense of guilt in using of commercial baby foods but choosing baby food labelled as organic was described as making them feel they had made a suitable choice. Others followed a more pragmatic approach using everyday family foods where food choices were justified by opportunity, what others were doing and trusting mother’s own instinct as to what their baby might like.

So with the baby food like they say organic, you don’t know if it is organic. They say that this is in there but it might not be in there and they say that like these fresh veggies but it might be like old, you just don’t know. It’s like hard to trust so you’d rather trust your own cooking than a jar of baby food.

*(FG4 Participant 4)*

Introducing new foods, textures and judging appropriate amounts was considered challenging and confusing. Mothers had difficulty in judging what textures to provide as their child grew and in judging the food photos of different textures used their own experiences as a framework. Age specific commercial baby food was chosen in the belief that the texture was age-appropriate and would reduce the likelihood of choking, which was a fear that permeated through discussions.

Just goes to show then no two are the same are they?

*(FG3 Participant 1)*

#### 3.3.4. Theme 4: *It’s not a Sin to Start them too Early or too Late*—Guidelines are Advice not Requirements

There was general consensus that government guidelines were based on research done on mothers who know a lot about children and that they are based more on opinion than a scientific basis and therefore to be used as an additional form of advice as opposed to a set of health recommendations to be adhered to at all costs.

I think they need to set guidelines to let people know that it’s ok to vary it from the guidelines. That it’s not a sin to start them too early or too late.

*(FG5- Participant 1)*

Part of the tensions in the interviews with ‘guidelines’ or ‘recommendations’ were when mothers own experiences and their mothering instincts didn’t fit with the AIFG. This was justified with statements that the guidelines’ around six months age target was overly cautious.

Follow like the guidelines you know what they should eat at what stage but then I guess because seeing her, what she was capable of eating, from there I just made up my own mind and like you know and what she could handle so I kind of just, the guidelines went out the window for me.

*(FG4-Participant 3)*

##### Subtheme—Role of Child Health Nurses

Mothers talked about the variable advice given by child health nurses having their *‘own take’* (FG7 Participant 1) on the age for introduction, even when the women knew the guidelines for the introduction of solid food was around six months. There was some confusion around the ideal age to start solid food, for these women the four month age milestone appeared to signal a mother’s assessment that their baby was showing developmental signs of readiness.

They just basically tell you when the child’s ready that’s when you put your child on solids and there’s not any specific guidelines, they just look at as every child’s different sort of thing.

*(FG6-Participant 3)*

We’ve got a child health nurse and they go the guideline says 6 months but we kind of know that it’s really 4, 4½ months where they start showing signs that they’re ready. You need to pay attention to the signs.

*(FG2-Participant 1)*

##### Subtheme Confusing Age Guidelines

The availability of commercial baby food with 4 months on the packaging confirmed this as the starting age for a number of women that contributed to further confusion as to the recommended starting age.

‘cause I’ve never had any guidelines, I’ve never been to see a child health nurse, I was told by a doctor that I need to do that so I’ve no idea. So I just go, I see it in the shop it says 4 months and I’m sweet, she’s 4 months, done.

*(FG5-Participant 5)*

Of concern from these discussions were that health professionals were considered to be always changing their advice based on the perception (incorrect) of some mothers that the new guidelines allowed for earlier introduction of solid food than the previous guidelines.

(Guidelines)… change so regularly they’re all just forever going back and forth on what it should be so you can’t really take too much with that.

*(FG4-Participant1)*

## 4. Discussion

The findings from this study suggest that adopting recommended practices related to the introduction of solid food continues to be difficult for mothers in Australia. This research was conducted at a time when the recommendation that solid food be introduced at around six months had been in place since 2003 and was reinforced in the latest revision of the AIFG published in 2012, several years before this study [2]. Our results have demonstrated a number of themes and subthemes on how feeding babies is constructed and justified. The results confirm those from the two systematic reviews of qualitative research and reinforce that misjudging cues for readiness, receiving multiple and conflicting advice and trusting own instincts in being a good mother continue to influence infant feeding practices [12,14]. They are also in keeping with international studies [35,36] that indicate mothers see the introduction of solid food as a major developmental milestone, which they are keen for their infant to attain. In spite of all this research, there is still an acknowledged gap between government guidelines such as the AIFG and the reality of introduction of solid food in practice.

### 4.1. Signs of Readiness

Mothers need more education on what are the true signs of readiness [37,38] as in this study they appeared to misjudge or be unsure of the signs of readiness. This was evidenced in the variable descriptions of how the actual first day of solid food eventuated. The social construction of babies as autonomous or agentive children [39] and able to decide when they needed solid food was the primary reason for early introduction [6]. This level of baby autonomy has been used to justify the early introduction as being the ‘right’ age by women in the UK [40]. Research exploring the communication between mothers and babies described a positive feedback pattern between mother and baby but that this resulted from inaccurate interpretation by mothers of infant cues that in turn lead to inappropriate introduction of solid food [41].

There is very little research on baby-led weaning in Australia but it is apparent from our results that the term baby-led weaning (BLW) has entered the lexicon of Australian mothers. This acknowledgement may stem from expectations of how feeding should progress [39]. However, interpretation of BLW by women in this study related mainly to what they perceived to be the signs of readiness for solid food on the part of the baby and not on the types of foods to be consumed. Even amongst those who purported to be using a BLW approach, only one mother identified practices that were consistent with the definition of BLW [42,43]. Mothers considered finger foods to be an example of BLW that they combined with spoon-feeding. A study of mothers in New Zealand [43] revealed that while roughly one third purported to be following BLW practices, one in five mothers were using spoon-feeding at least half of the time. Only one in ten mothers adhered strictly to BLW principles [44] whereby the infant had complete control over their own eating from the beginning of the introduction of complementary foods.

Our mothers universally did not think about the introduction of solid food prior to their baby’s birth. This is similar to comments in interviews with Australian pregnant women that few had considered the introduction of complementary food into their child’s diet [10]. Nutrition reasons and health risks related to the age of introduction of solid food were not the focus of mothers in this study when deciding when to introduce solid food. There may exist a difference in the health promotion messaging of the importance of nutrition between different countries as research with Danish women found nutrition was a key focus in the decision related to the introduction of solid food [45]. 

### 4.2. Sources of Information

The multiple but conflicting sources of information identified in this research are similar to those identified in recent studies in Australia [16,46] the United Kingdom [40] and United States [47]. Women say they were more likely to seek information about infant feeding options from people they perceive have experience, rather than from health professionals such as nurses and doctors. Women appreciated anecdotes from friends about their experiences and the encouragement and support friends provided on a day-to-day basis [10,48]. Personal advice from sources close to the mother tended to lead to earlier introduction of solid food than professional advice and may explain some differences between low and higher socioeconomic groups [49]. This is consistent with the findings of a recent systematic review that concluded conflicting advice leads women to adopt practices that are valued by the family or culturally established practices [12]. Health professionals need to seek all opportunities to promote infant feeding guidelines and to do this in a way that positively engages parents in their new roles and builds confidence to override family advice and cultural expectations.

### 4.3. Being a Good Mother

In conjunction with a lack of prior thought about the introduction of solid food, mothers are adjusting to becoming a mother and continuously developing their identity [50]. Infant feeding guidelines such as those related to the introduction of solid food assume a rational and informed decision-making process; however, there is a need to consider that it is not just a cognitive process but also an emotional process [51]. Whilst a mother’s intention may be to follow official advice, the concept identified in this and other studies that every baby is different is used to justify the reality of everyday lives and need for flexibility [17,48]. Mothers justify their interpretations of when their baby was ready not only by the age of their baby but also by a range of other indicators, which are not always appropriately assessed [12]. 

The introduction of solid food was an important milestone for mothers in this research but there was an *ad hoc* approach in practice, described in other research as introducing solid food for the fun of it [38]. Of concern was the peer pressure that influenced the early introduction of solid food, and other studies have found that competition to have a baby at an advanced stage of development may encourage mothers to introduce solid food early [37]. Even parents who have persevered with their ideals may reach a pivotal point when they introduce formula or solid food earlier than planned as a coping strategy [12] based on myths of immediate gain of a more settled and contented baby, relief from their anxiety and more sleep [17,38,52].

Mothers need to negotiate the commercial baby food market and associated advertising. Commercial baby food manufacturers are seen to market mass produced food with messaging to indicate homemade or safe including an emphasis on organic [53]. There are tensions in considering the risk of using these foods and the guilt mothers may feel that their own cooking is not good enough. Baby food advertising questions the mother’s ability and rationale for home cooking. This is achieved by drawing upon a common advertising gimmick that new babies are unfathomable and new parents exist in a state of chaos bending to the demands of their infant [54] and plays on the convenience factor [55]. In general, mothers in this study discussed commercial baby foods negatively and this is confirmed in similar studies [35]. However, the presence of these foods in the supermarket does influence practices [56] and the labelling of foods as suitable from four months of age that persists in Australia undermines the AIFG and causes confusion amongst mothers [57]. There is very little research on the food skills, particularly cooking skills of new mothers and how this might be related to food choices for babies and this is an area for future research.

### 4.4. Role of Infant Feeding Guidelines

There is a need to review communication and education messaging as expectations that infant feeding guidelines will results in parents taking morally responsible actions is not translating into practice [58]. Our results demonstrated a lack of knowledge of and understanding of how the AIFG are developed and the rationale for age guidelines. The lack of awareness of the government guidelines and some reported trust issues with the content raise questions for how the guidelines are translated and promoted to parents. Similar trust issues have been reported in other countries for example in the UK [59] where even when viewed as official they were dismissed if they did not fit the context for individual mothers, babies and families. In addition, because mothers did not perceive them to be based on scientific evidence they did not explicitly discuss risk of too early or too late introduction and/or concerns around food allergy and obesity development. Any risks of the early introduction of solid food certainly were downplayed or considered non-existent [16] in order to justify perceived benefits of early introduction for mothers, babies and families. 

Health professionals need to think how to communicate guidelines to parents to empower them to avoid the early introduction solid food to overcome barriers such as the perceived need feed to sleep through the night. Infant feeding guidelines were perceived as general advice that mothers interpret and adjust to suit individual differences in babies. This produces a situation where there is conflict and tensions between the advice by governments and health professionals and the reality of feeding babies, which in turn challenges mothers’ identities. Hoddinott [52] refers to this contradiction as idealism vs realism. Dealing with this contradiction sees a trade-off between the two and studies report whilst mothers seek advice on feeding from health care professionals they adapt this to the needs of their infant [16,60]. Making an informed decision as a parent comes with judgement from those seen with expert knowledge. A recent review of the practice of nurses in UK and Canada has cautioned health professionals to be aware how their expert positions guide the type of support and care given to mothers and the need to acknowledge different ways of knowing about infant feeding [61].

The strengths of this study is that it is the first qualitative study for Western Australia and provides additional high quality evidence for the Australian context. The use of focus groups enabled group interactions on the issue of concern and uncovered a range of knowledge and experiences. Focus groups were conducted until no new relevant knowledge was obtained. In considering the transferability of the results, participants were a purposeful sample recruited from existing groups in disadvantaged areas and may not represent the population of mothers. Our participants were similar age and had slightly higher education that the general population of childbearing women aged 20–40 but not as educated as other research with two thirds of their sample with tertiary qualifications [17,18]. We only recruited from a metropolitan area and in 21.2% of babies were born in regional areas [33].

## 5. Conclusions

This study demonstrates the continued issues in achieving the infant feeding guidelines in developed countries like Australia. Infant feeding guidelines are one of a number of considerations mothers are juggling at the time of introducing solid food. The introduction of solid food is a significant transition in the dietary intake of infants and a potential time of peer pressure and confusion for mothers. Health professionals have a role to provide mothers with detail on developmental signs of readiness, texture and food changes and nutrition reasons for the age recommendation. More research is required on effective education strategies for parents. Health professionals must address barriers and support enablers to achieving infant feeding recommendations in the design of nutrition intervention programs and education materials for parents and families, particularly given the conflicting advice women report at this time. Our research will assist health professionals giving advice to support the implementation of the infant feeding guidelines, particularly in trying to move the accepted age from four months to closer to the recommended around six months.

## Figures and Tables

**Table 1:** Characteristics of focus group participants (n = 42).

Demographic Characteristics	n	%
Mother’s age (yrs)		
<25	9	21.4
25–29	11	26.2
30–34	11	26.2
35+	11	26.2
Mother’s level of education		
<10 yrs	2	4.8
Year 10 or equivalent	1	2.4
Year 12 or equivalent	6	14.3
Trade/diploma	13	31.0
Bachelor degree or higher	18	42.9
Missing	2	4.8
Infant age (months)		
<6	6	14.3
6–8	13	31.0
9–11	11	26.2
≥ 12	12	28.6
Age solid food introduced (months)		
<4	5	11.9
4 to <6	29	69.0
≥6	8	19.0
Age stopped breastfeeding (months) (n = 22)		
<2	8	36.4
2–3	6	27.3
4–5	3	13.6
≥ 6	5	22.7
Age received formula (months) (n = 29)		
<4	25	59.5
4 to <6	3	7.1
≥6	14	33.3

## References

[1] National Health and Medical Research Council (2003). Dietary Guidelines for Australian Adults.

[2] National Health and Medical Research Council (2012). Infant Feeding Guidelines.

[3] Donath S.M., Amir L.H. (2005). Breastfeeding and the introduction of solids in australian infants: Data from the 2001 national health survey. Aust. N. Z. J. Public Health.

[4] Scott J.A., Binns C.W., Graham K.I., Oddy W.H. (2009). Predictors of the early introduction of solid foods in infants: Results of a cohort study. BMC Pediatr..

[5] Australian Institute of Health and Welfare (2011). 2010 Australian National Infant Feeding Survey: Indicator Results.

[6] Newby R., Davies P. (2015). A prospective study on the introduction of complementary foods in comtemporary australian infants: What, when and why?. J. Paediatr. Child Health.

[7] Wen L.M., Baur L.A., Rissel C., Alperstein G., Simpson J.M. (2009). Intention to breastfeed and awareness of health recommendations: Findings from first-time mothers in southwest sydney, australia. Int. Breastfeed. J..

[8] Lupton D.A. (2011). ‘The best thing for the baby’: Mothers’ concepts and experiences related to promoting their infants’ health and development. Health Risk Soc..

[9] Hauck Y.L., Graham-Smith C., McInerney J., Kay S. (2011). Western australian women’s perceptions of conflicting advice around breast feeding. Midwifery.

[10] York E., Hoban E. (2013). Infant feeding intentions among first time pregnant women in urban melbourne, australia. Midwifery.

[11] Brouwer M.A., Drummond C., Willis E. (2012). Using goffman’s theories of social interaction to reflect first-time mothers’ experiences with the social norms of infant feeding. Qual. Health Res..

[12] Harrison M., Brodribb W., Hepworth J. (2017). A qualitative systematic review of maternal infant feeding practices in transitioning from milk feeds to family foods. Matern. Child Nutr..

[13] Zehle K., Wen L.M., Orr N., Rissel C. (2007). “It’s not an issue at the moment”: A qualitative study of mothers about childhood obesity. MCN Am. J. Matern. Child Nurs..

[14] Matvienko-Sikar K., Kelly C., Sinnott C., McSharry J., Houghton C., Heary C., Toomey E., Byrne M., Kearney P.M. (2018). Parental experiences and perceptions of infant complementary feeding: A qualitative evidence synthesis. Obes. Rev..

[15] Kuswara K., Laws R., Kremer P., Hesketh K.D., Campbell K.J. (2016). The infant feeding practices of chinese immigrant mothers in australia: A qualitative exploration. Appetite.

[16] Russell C.G., Taki S., Azadi L., Campbell K.J., Laws R., Elliott R., Denney-Wilson E. (2016). A qualitative study of the infant feeding beliefs and behaviours of mothers with low educational attainment. BMC Pediatr..

[17] Walsh A., Kearney L., Dennis N. (2015). Factors influencing first-time mothers’ introduction of complementary foods: A qualitative exploration. BMC Public Health.

[18] Spence A.C., Hesketh K.D., Crawford D.A., Campbell K.J. (2016). Mothers’ perceptions of the influences on their child feeding practices—A qualitative study. Appetite.

[19] Harrison M., Hepworth J., Brodribb W. (2018). Navigating motherhood and maternal transitional infant feeding: Learnings for health professionals. Appetite.

[20] Baillie L. (2015). Promoting and evaluating scientific rigour in qualitative research. Nurs. Stand..

[21] Tong A., Sainsbury P., Craig J. (2007). Consolidated criteria for reporting qualitative research (coreq): A 32-item checklist for interviews and focus groups. Int. J. Qual. Health Care.

[22] Murphy E. (2000). Risk, responsiblity and rhetoric in infant feeding. J. Contemp. Ethnogr..

[23] Bisogni C.A., Jastran M., Seligson M., Thompson A. (2012). How people interpret healthy eating: Contributions of qualitative research. J. Nutr. Educ. Behav..

[24] Thomas D.R. (2016). A general inductive approach for analyzing qualitative evaluation data. Am. J. Eval..

[25] Draper A., Swift J.A. (2011). Qualitative research in nutrition and dietetics: Data collection issues. J. Hum. Nutr. Diet..

[26] Scott J., Binns C.W., Arnold R. (1997). Attitudes towards breastfeeding in Perth, Australia qualitative analysis. J. Nutr. Educ..

[27] Scott J.A., Mostyn T., Greater Glasgow Breastfeeding Initiative Management Team (2003). Women’s experiences of breastfeeding in a bottle-feeding culture. J. Hum. Lact..

[28] Tattersall C., Watts A., Vernon S. (2007). Mind mapping as a tool in qualitative research. Nurs. Times.

[29] Harper D. (2002). Talking about pictures: A case for photo elicitation. Vis. Stud..

[30] Saunders B., Sim J., Kingstone T., Baker S., Waterfield J., Bartlam B., Burroughs H., Jinks C. (2018). Saturation in qualitative research: Exploring its conceptualization and operationalization. Qual. Quant..

[31] Denzin N.K., Lincoln Y.S. (2011). The Sage Handbook of Qualitative Resarch.

[32] Miles M., Huberman A. (1994). Qualitative Data Analysis.

[33] Maternal and Child Health Unit (2016). Western Australia’s Mothers and Babies, 2013. 31st Annual Report of the Western Australian Midwives’ Notification System.

[34] Australian Bureau of Statistics Census of population and housing (2016), TableBuilder. Findings based on the use of the abs TableBuilder data. http://www.abs.gov.au/websitedbs/censushome.nsf/home/tablebuilder?opendocument&navpos=240.

[35] Schwartz C., Scholtens P.A., Lalanne A., Weenen H., Nicklaus S. (2011). Development of healthy eating habits early in life. Review of recent evidence and selected guidelines. Appetite.

[36] Anderson A.S., Guthrie C.A., Alder E.M., Forsyth S., Howie P.W., Williams F.L. (2001). Rattling the plate--reasons and rationales for early weaning. Health Educ. Res..

[37] Arden M.A. (2010). Conflicting influences on UK mothers’ decisions to introduce solid foods to their infants. Matern. Child Nutr..

[38] Brown A., Rowan H. (2016). Maternal and infant factors associated with reasons for introducing solid foods. Matern. Child Nutr..

[39] Locke A. (2015). Agency, ‘good motherhood’ and ‘a load of mush’: Constructions of baby-led weaning in the press. Women’s Stud. Int. Forum.

[40] Moore A.P., Milligan P., Goff L.M. (2014). An online survey of knowledge of the weaning guidelines, advice from health visitors and other factors that influence weaning timing in UK mothers. Matern. Child Nutr..

[41] Waller J., Bower K.M., Spence M., Kavanagh K.F. (2015). Using grounded theory methodology to conceptualize the mother-infant communication dynamic: Potential application to compliance with infant feeding recommendations. Matern. Child Nutr..

[42] Rapley G., Hall Moran V., Dykes F. (2006). Baby-led weating. A developmental approach to the introduction of complementary foods. Maternal and Infant Nutrition and Nuture. Controversies and Challenges.

[43] Cameron S.L., Taylor R.W., Heath A.L. (2013). Parent-led or baby-led? Associations between complementary feeding practices and health-related behaviours in a survey of New Zealand families. BMJ Open.

[44] Rapley G. (2011). Baby-led weaning: Transitioning to solid foods at the baby’s own pace. Community Pract..

[45] Nielsen A., Michaelsen K.F., Holm L. (2014). Beyond an assumed mother–child symbiosis in nutritional guidelines: The everyday reasoning behind complementary feeding decisions. Child Care Pract..

[46] Newby R., Brodribb W., Ware R.S., Davies P.S. (2015). Antenatal information sources for maternal and infant diet. Breastfeed. Rev..

[47] Ashida S., Lynn F.B., Williams N.A., Schafer E.J. (2015). Competing infant feeding information in mothers’ networks: Advice that supports v. Undermines clinical recommendations. Public Health Nutr..

[48] Caton S.J., Ahern S.M., Hetherington M.M. (2011). Vegetables by stealth. An exploratory study investigating the introduction of vegetables in the weaning period. Appetite.

[49] Gage H., Williams P., Von Rosen-Von Hoewel J., Laitinen K., Jakobik V., Martin-Bautista E., Schmid M., Egan B., Morgan J., Decsi T. (2012). Influences on infant feeding decisions of first-time mothers in five european countries. Eur. J. Clin. Nutr..

[50] Miller T. (2007). “Is this what motherhood is all about?” Weaving expriences and discourse through transition to first-time motherhood. Gend. Soc..

[51] Sheehan A., Schmied V., Barclay L. (2013). Exploring the process of women’s infant feeding decisions in the early postbirth period. Qual. Health Res..

[52] Hoddinott P., Craig L.C., Britten J., McInnes R.M. (2012). A serial qualitative interview study of infant feeding experiences: Idealism meets realism. BMJ Open.

[53] Moisio R., Arnould E., Price L. (2004). Between mothers and markets: Constructing family identify through homemade food. J. Consum. Cult..

[54] Davies A. (2006). Too many cooks spoil the broth? Mother’s authority on food and feeding. Advert. Soc. Rev..

[55] Fuentes M., Brembeck H. (2016). Best for baby? Framing weaning practice and motherhood in web-mediated marketing. Consum. Markets Cult..

[56] Hamilton K., Daniels L., Murray N., White K.M., Walsh A. (2012). Mothers’ perceptions of introducing solids to their infant at six months of age: Identifying critical belief-based targets to promote adherence to current infant feeding guidelines. J. Health Psychol..

[57] Dunford E., Louie J.C., Byrne R., Walker K.Z., Flood V.M. (2015). The nutritional profile of baby and toddler food products sold in australian supermarkets. Matern. Child Health J..

[58] Knaak S.J. (2010). Contextualising risk, constructing choice: Breastfeeding and good mothering in risk society. Health Risk Soc..

[59] O’Key V., Hugh-Jones S. (2010). I don’t need anybody to tell me what I should be doing. A discursive analysis of maternal accounts of (mis)trust of healthy eating information. Appetite.

[60] Synnott K., Bogue J., Edwards C.A., Scott J.A., Higgins S., Norin E., Frias D., Amarri S., Adam R. (2007). Parental perceptions of feeding practices in five european countries: An exploratory study. Eur. J. Clin. Nutr..

[61] Peckover S., Aston M. (2018). Examining the social construction of surveillance: A critical issue for health visitors and public health nurses working with mothers and children. J. Clin. Nurs..

